# Verbal Memory Deficits Are Correlated with Prefrontal Hypometabolism in ^18^FDG PET of Recreational MDMA Users

**DOI:** 10.1371/journal.pone.0061234

**Published:** 2013-04-09

**Authors:** Oliver G. Bosch, Michael Wagner, Frank Jessen, Kai-Uwe Kühn, Alexius Joe, Erich Seifritz, Wolfgang Maier, Hans-Jürgen Biersack, Boris B. Quednow

**Affiliations:** 1 Department of Psychiatry, Psychotherapy, and Psychosomatics, University Hospital of Psychiatry, Zurich, Switzerland; 2 Department of Psychiatry and Psychotherapy, University of Bonn, Bonn, Germany; 3 German Center for Neurodegenerative Disorders (DZNE), Bonn, Germany; 4 Department of Psychiatry, Saarland University Medical Center, Homburg, Germany; 5 Department of Nuclear Medicine, University of Bonn, Bonn, Germany; 6 Zurich Center for Integrative Human Physiology, University of Zurich, Zurich, Switzerland; University G. D'Annunzio, Italy

## Abstract

**Introduction:**

3,4-Methylenedioxymethamphetamine (MDMA, “ecstasy”) is a recreational club drug with supposed neurotoxic effects selectively on the serotonin system. MDMA users consistently exhibit memory dysfunction but there is an ongoing debate if these deficits are induced mainly by alterations in the prefrontal or mediotemporal cortex, especially the hippocampus. Thus, we investigated the relation of verbal memory deficits with alterations of regional cerebral brain glucose metabolism (rMRGlu) in recreational MDMA users.

**Methods:**

Brain glucose metabolism in rest was assessed using 2-deoxy-2-(^18^F)fluoro-D-glucose positron emission tomography (^18^FDG PET) in 19 male recreational users of MDMA and 19 male drug-naïve controls. ^18^FDG PET data were correlated with memory performance assessed with a German version of the Rey Auditory Verbal Learning Test.

**Results:**

As previously shown, MDMA users showed significant impairment in verbal declarative memory performance. PET scans revealed significantly decreased rMRGlu in the bilateral dorsolateral prefrontal and inferior parietal cortex, bilateral thalamus, right hippocampus, right precuneus, right cerebellum, and pons (at the level of raphe nuclei) of MDMA users. Among MDMA users, learning and recall were positively correlated with rMRGlu predominantly in bilateral frontal and parietal brain regions, while recognition was additionally related to rMRGlu in the right mediotemporal and bihemispheric lateral temporal cortex. Moreover, cumulative lifetime dose of MDMA was negatively correlated with rMRGlu in the left dorsolateral and bilateral orbital and medial PFC, left inferior parietal and right lateral temporal cortex.

**Conclusions:**

Verbal learning and recall deficits of recreational MDMA users are correlated with glucose hypometabolism in prefrontal and parietal cortex, while word recognition was additionally correlated with mediotemporal hypometabolism. We conclude that memory deficits of MDMA users arise from combined fronto-parieto-mediotemporal dysfunction.

## Introduction

3,4-Methylenedioxymethamphetamine (MDMA, “ecstasy”) is an illicit club drug predominantly used by adolescents and young adults for its euphoric, stimulant, and empathogenic/entactogenic properties [1], [2]. After cannabis and cocaine, MDMA together with other amphetamines are the most commonly used illegal drugs in Europe and worldwide [3], [4]. In a recent survey, 5.5% of the European general population between 15 to 34 years of age have taken ecstasy at least once, with an estimated number of 7.5 million users [3].

The psychotropic effects of MDMA are mediated primarily through reversal and inhibition of the serotonin (5-HT) transporter (5-HTT) [5] leading to a significant increase of 5-HT in the synaptic cleft [6]. Other central effects include increases of extracellular dopamine concentrations in the striatum and the prefrontal cortex [7], glutamate release in the hippocampus [8], an a massive elevation of peripheral cortisol levels of 150–800% [9].

Numerous animal studies with different species provided compelling evidence for an impairment of the 5-HT system following MDMA exposure [6], . In non-human primates, MDMA-induced loss of uniquely 5-HT nerve terminals with a concomitant depletion of up to 95% of 5-HT was observed predominantly in frontal and mediotemporal cortical regions, while other monoamine neurotransmitters such as dopamine or norepinephrine remained unaffected [12]–[16]. Early studies already suggested an impairment of the 5-HT system in human MDMA users because they display decreased cerebrospinal fluid (CSF) levels of the 5-HT metabolite 5-hydroxyindoleacetic acid (5-HIAA)[17], [18]. The human neurotoxicity of MDMA was also supported by the highly consistent finding that at least intense MDMA users display marked verbal and visuo-spatial memory deficits [19]–[27]. Importantly, lowered 5-HIAA CSF levels in MDMA users have been shown to be correlated with memory deficits [25], [28]. Finally, also electrophysiological studies suggested alterations of the 5-HT system in regular MDMA users [29]–[31].

Human imaging studies using 2-deoxy-2-(^18^F)fluoro-D-glucose (^18^FDG) positron emission tomography (PET) showed reduced metabolism in the left hippocampus [32], in bilateral caudate/putamen, the left amygdala [33], [34], and in the dorsolateral prefrontal cortex (DLPFC) [35] of MDMA users. Finally, in a large sample of MDMA users, increased glucose metabolism in the ventrolateral frontal cortex (Brodman areal [BA] 10) was reported as well [33].

PET studies using serotonergic radioligands consistently showed globally reduced 5-HTT binding in chronic MDMA users most pronounced in mediotemporal and frontal cortices [36]–[41]. These findings were confirmed recently, as widespread cortical and striatal reductions in 5-HTT levels were shown in MDMA users [42]. In this sample, time of abstinence was positively correlated with subcortical, but not cortical, 5-HTT binding, suggesting only a partial neuronal recovery in these subjects. However, another recent study failed to find alterations of 5-HTT binding in recreational MDMA users [43]. Also investigations of postsynaptic 5-HT receptors revealed rather inconsistent results. While early SPECT studies demonstrated decreased cortical 5-HT_2A_ receptor binding in current MDMA users and an increase of 5-HT_2A_ receptors in former users [44], recent PET studies reported either a small decrease [42] or an increase of cortical 5-HT_2A_ receptor density in current MDMA users [45], [46].

Interpreting the neuropsychological profile, it was speculated that MDMA users have deficits during storage and/or retrieval of memory information arising from a dysfunction of the mediotemporal cortex [20], [21], [47]. Functional magnetic resonance imaging (fMRI) studies also suggested differences in the activation of the hippocampus between MDMA users and healthy controls during working memory tasks and supported the view of a hippocampal dysfunction in MDMA users [48]–[52]. However, further functional imaging studies additionally reported abnormalities in the activation of several other brain regions–including frontal, thalamic, striatal, cingulate, temporolateral, parietal, and occipital regions–of MDMA users during working memory tasks [50], [52]–[54]. Neuropsychological evidence additionally points to an MDMA-related impairment of executive functions such as impulsivity, decision-making, and recall consistency indicating frontal dysfunction [27], [55], [56].

Thus, although memory deficits are the most consistent finding in MDMA users [57], their neurobiological basis are unclear so far. Moreover, no study explored cerebral glucose metabolism assessed by PET in relation to MDMA-related memory deficits subdivided into different functions such as learning, recall, and recognition. Therefore, we investigated regional cerebral glucose metabolism (rMRGlu) and verbal memory performance of recreational MDMA users and drug-naïve controls by ^18^FDG PET and the Rey Auditory Verbal Learning Test (RAVLT). Subsequently, we correlated rMRGlu with several RAVLT parameters in both groups separately to determine the origin of memory dysfunction in MDMA users. The neuropsychological pattern already published from this sample suggested that frontal and mediotemporal dysfunction might be involved in the development of memory deficits [27]. Thus, we expect to find correlations specifically between decreased rMRGlu in frontal and temporomedial brain regions and diminished memory performance in MDMA users.

## Results

### Demographics and drug use

Both groups did not significantly differ with respect to age, handedness, years of education, and verbal intellectual performance (**Table 1**). There were fewer smokers in the drug-naïve control group compared to the MDMA group and mean cigarettes per day also differed between both groups (MDMA: 11.6±8.6 SD; controls: 3.1±8.0 SD; T(36) = 3.19, p<.003). The amount of illicit drug use is presented in **Table 2**. Importantly, the MDMA group used predominantly MDMA, while the use of amphetamine, cocaine, and hallucinogens was only sporadic. However, MDMA users also revealed a mild to moderate cannabis use. In contrast, drug-naïve controls did not report any experiences with illicit drugs.

**Table 1:** Demographic data of 19 male recreational MDMA users and 19 male drug-naïve controls (numbers or means and standard deviations in parentheses).

	Total	MDMA users	Controls	Value^a^	df	*p*
**N**	38	19	19			
**Age**	23.8 (5.0)	24.2 (5.8)	23.4 (4.3)	*T* = 0.48	36	0.64
**Handedness right/left**	32/6	16/3	16/3	*χ* ^2^ = 0.00	1	1.0
**Smoker/Nonsmoker**	20/18	15/4	5/14	*χ* ^2^ = 8.55	1	0.004
**Verbal IQ**	103.2 (12.7)	100.6 (11.7)	105.7 (13.5)	*T* = −1.25	36	0.22
**Years of education**	12.4 (1.6)	12.3 (1.7)	12.5 (1.5)	*T* = −0.30	36	0.77

a T-tests or Chi^2^-test (with Yates correction) for frequency data.

**Table 2:** Pattern and amount of illegal drug use: results of the Psychotropic Drug Interview (means and standard deviations in parentheses).

Drug characteristics^a^		MDMA users	Controls
**MDMA**	Tablets per week	1.97 (2.73)	0.00 (0.00)
	Years of use	3.66 (1.95)	0.00 (0.00)
	Cumulative dose (tablets)	457.9 (433.9)	0.00 (0.00)
	Lifetime peak dose^b^ (tablets)	6.1 (4.7)	0.00 (0.00)
	Last consumption (days)	17.4 (14.6); *n = 19*	0.00 (0.00)
**Cannabis**	Times per week	1.63 (1.62)	0.00 (0.00)
	Years of use	3.95 (3.11)	0.00 (0.00)
	Cumulative dose (times)	547.1 (502.7)	0.00 (0.00)
	Last consumption (days)	11.1 (21.6); *n = 16*	0.00 (0.00)
**Amphetamine**	Times per week	0.82 (1.31)	0.00 (0.00)
	Years of use	3.37 (2.05)	0.00 (0.00)
	Cumulative dose (times)	208.5 (279.5)	0.00 (0.00)
	Last consumption (days)	38.1 (89.9); *n = 17*	0.00 (0.00)
**Cocaine**	Times per week	0.04 (0.10)	0.00 (0.00)
	Years of use	0.66 (1.70)	0.00 (0.00)
	Cumulative dose (times)	4.87 (12.51)	0.00 (0.00)
	Last consumption (days)	34.5 (17.2); *n = 4*	0.00 (0.00)
**Hallucinogens** c	Cumulative dose (times)	23.4 (38.8)	0.00 (0.00)
	Last consumption (month)	7.86 (9.37); *n = 14*	0.00 (0.00)

a Consumption per week, duration of use, and cumulative dose are averaged within the total group. Last consumption is averaged only for persons who used the drug. In this case, sample size, *n*, is shown

b Highest single MDMA dose ever used.

c Primarily LSD and psilocybin-containing mushrooms were used.

### Verbal Memory

As reported previously from this sample [27], immediate (supraspan, trial 1) and delayed recall (trial 7), learning performance (Σ trials 1–5), and recall consistency from the RAVLT was significantly worse in MDMA users compared to controls with medium to large effect sizes (d = 0.66–1.13, **Table 3**). Moreover, MDMA users revealed a slight and non-significant reduction of verbal recognition, showing, however, a medium effect size. Previously, we already demonstrated that several indicators of MDMA use were significantly correlated with memory scores [27].

**Table 3:** Performance in the Rey Auditory Verbal Learning Task (RAVLT) of MDMA users and healthy drug-naive controls (means and standard deviations in parentheses).

	MDMA users	Controls	T	df	p	Cohen's d
**Supraspan** (*trial 1*)	8.1 (2.3)	9.6 (2.0)	−2.11	36	0.042	0.66
**Learning performance** (*Σ trials 1*–*5*)	56.2 (8.2)	64.7 (5.7)	−3.71	36	0.001	1.04
**Delayed recall** (*trial 7*)	11.2 (2.9)	14.2 (1.0)	−4.21	36	0.0002	1.13
**Recall consistency, trials 1–5** (*in percent*)	86.6 (8.6)	95.1 (4.8)	−3.77	36	0.001	1.05
**Adjusted recognition performance list A** (*p*(*A*))	0.85 (0.1)	0.90 (0.1)	−1.68	36	0.102	0.53

### Brain metabolism

**Figure 1:**
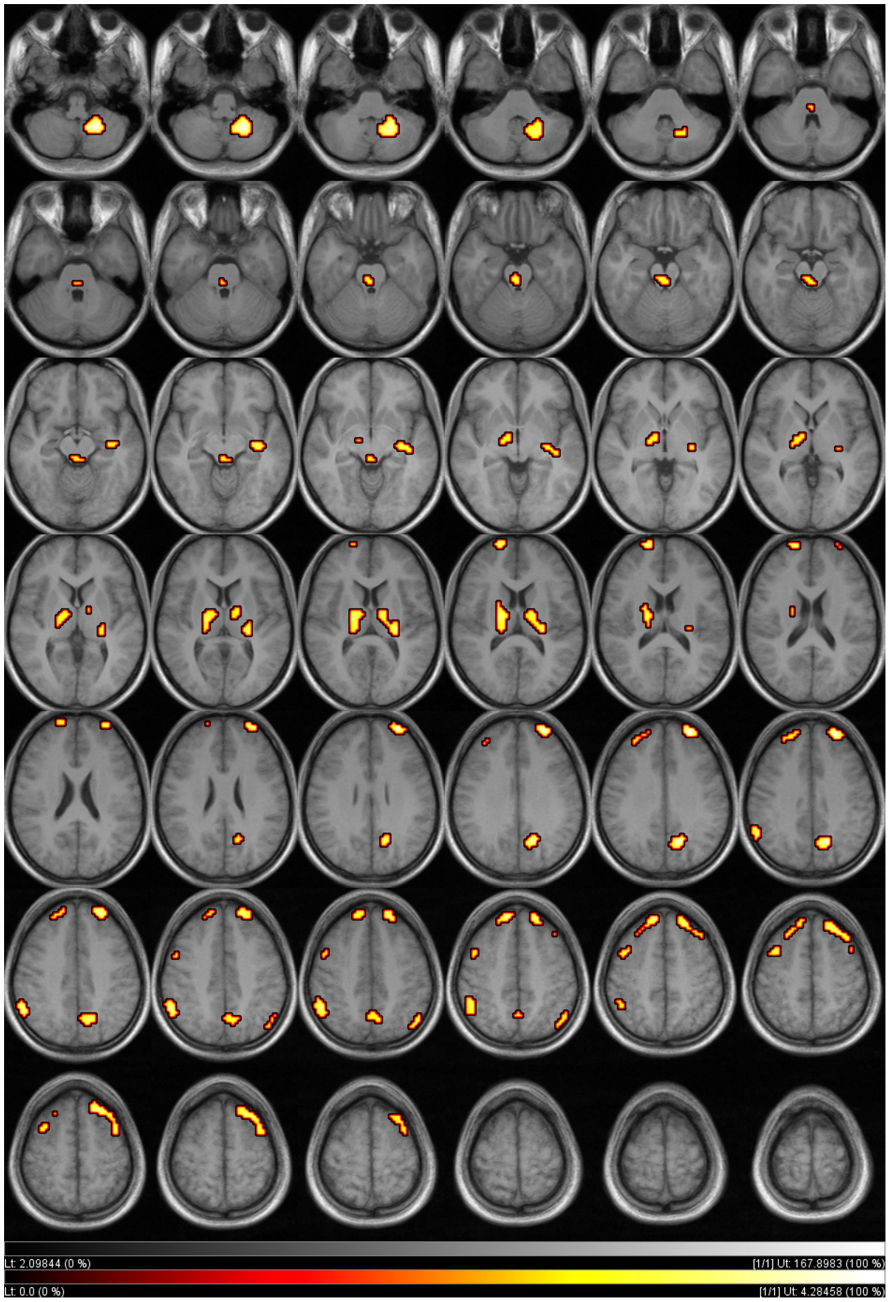
Brain regions in which regional glucose metabolism was significantly decreased in 19 recreational MDMA users compared to 19 drug-naïve healthy controls (p<.005, uncorrected, cluster level >25 voxel, clusters projected on an SPM MRI template).

MDMA users showed a significant decrease of resting rMRGlu in the right and left DLPFC (**Figure 1** **,** **Table 4**). Bilateral frontal hypometabolism ranged from BA 8, 9, and 10 up to the premotor cortex (BA 6), showing slightly stronger effects in the right hemisphere (**Figure 1** **,** **Table 4**). Moreover, a significant decrease of resting rMRGlu in MDMA users could be demonstrated for the bilateral inferior parietal cortex (BA 40), as well as in the right precuneus (BA 7). In addition, the bilateral thalamus showed significant hypometabolism primarily in areas connected with the prefrontal cortex (PFC) [58]. The significant cluster including the right thalamus extended to the right hippocampus. A significant decrease of rMRGlu could also be shown in the metencephalon (pons) and mesencephalon (at the level of the rostral raphe nuclei) as well as in the right posterior cerebellum of the MDMA users (**Figure 1**). Increases of rMRGlu in MDMA users were not significant.

**Table 4:** Brain regions with significant decreased regional glucose metabolism in 19 recreational MDMA users compared to 19 drug-naïve controls (MNI coordinates of maximum significant voxels, p<.005, uncorrected).

*z (df 36)*	Cluster size	MNI coordinates			P_uncorrected_		Hemisphere		Anatomical region	Brodmann area
	*Voxel*	*x,y,z (mm)*			*voxel-level*					
3.83	257^a^	24	54	33		0.000		right	middle/superior frontal gyrus	8/9
3.77	114^b^	15	−63	33		0.000		right	precuneus	7
3.71	139^b^	21	−54	−51		0.000		right	cerebellum, posterior lobe	-
3.44	85	−57	−51	42		0.000		left	inferior parietal lobule	40
3.42	85	−12	45	48		0.000		left	middle/superior frontal gyrus	8/9
3.37	34	−18	66	15		0.000		left	superior frontal gyrus	10
3.24	134^b^	27	−24	9		0.001		right	thalamus (prefrontal), hippocampus	-
3.22	148^b^	−21	−15	9		0.001		left	thalamus (prefrontal)	-
3.15	25	48	−66	45		0.001		right	inferior parietal lobule	40
3.05	36	−39	9	54		0.001		left	middle frontal gyrus	6
2.98	63	−3	−30	−24		0.001		left	pons, raphe nuclei	-

a *Cluster significant at p<.05 (corrected for multiple testing).*

b *Cluster significant at p<.05 (uncorrected).*

### Correlations of brain metabolism with verbal memory deficits

**Figure 2:**
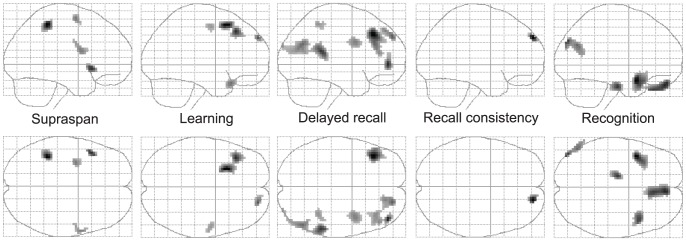
Brain regions in which decreased regional glucose metabolism was significantly correlated with low memory performance in 19 recreational MDMA users (p<.005, uncorrected, cluster level >25 voxel).

To investigate the relation of decreased resting rMRGlu with memory impairment we only investigated positive correlations between rMRGlu and memory performance (low performance in conjunction with decreased rMRGlu) in MDMA users (**Figure 2** **and** **Table 5**).

**Table 5:** Brain regions in which regional glucose metabolism was positively correlated with memory parameters in 19 recreational MDMA users (MNI coordinates of maximum significant voxels, p<.005, uncorrected).

*z (df 17)*	Cluster size	MNI coordinates			P_uncorrected_		Hemisphere		Anatomical region	Brodmann area
	*Voxel*	*x,y,z (mm)*			*voxel-level*					
***Supraspan***										
3.74	56	−39	−42	54		0.000		left	inferior parietal lobule	40
3.44	30	−45	18	−6		0.000		left	inferior frontal gyrus	47
2.99	31	−33	−3	63		0.001		left	middle frontal gyrus	8
2.98	32	60	3	18		0.001		right	precentral / inferior frontal gyrus	6/44
***Learning performance***										
4.42	129^a^	−24	15	54		0.000		left	middle frontal gyrus	8
3.65	25	21	57	36		0.000		right	superior frontal gyrus	9
3.40	49	−27	21	−24		0.000		left	inferior frontal gyrus	47
2.97	28	54	−6	51		0.001		right	precentral gyrus	6
***Delayed recall***										
4.45	140^a^	−42	30	39		0.000		left	middle frontal gyrus	8/9
4.06	56	45	48	0		0.000		right	inferior frontal gyrus	10
3.95	235^b^	57	−42	18		0.000		right	superior temporal gyrus	13
3.60	139^a^	21	54	36		0.000		right	superior frontal gyrus	9
3.27	70	39	3	30		0.001		right	precentral gyrus	6
3.08	36	36	−45	54		0.001		right	inferior parietal lobule	40
***Recall consistency***										
3.67	30	18	57	30		0.000		right	superior frontal gyrus	9
***Recognition***										
4.09	140^a^	−42	9	−21		0.000		left	superior/middle temporal gyrus	38/21
3.78	153^a^	6	48	−27		0.000		right	gyrus rectus	11
3.66	51	−20	−15	−30		0.000		left	parahippocampal gyrus/hippocampus	28
3.66	82	−45	−81	33		0.000		left	angular gyrus	39
3.57	94^a^	45	12	−24		0.000		right	superior temporal gyrus	38

a *Cluster significant at p<.05 (uncorrected).*

b *Cluster significant at p<.05 (corrected for multiple testing).*

#### Supraspan

Within the MDMA-group, low rMRGlu in the left inferior parietal (BA 40), left premotor (BA 6), left dorsolateral (BA 8), and left ventrolateral frontal cortex (BA 44, 47) was significantly correlated with worse performance during the first RAVLT trial (supraspan).

#### Learning

Within the MDMA-group, rMRGlu in the right premotor (BA 6), bilateral dorsolateral prefrontal (BA 8, 9), and the left inferior frontal cortex (BA 47) was significantly correlated with the total learning performance in the RAVLT.

#### Delayed recall

Within the MDMA-group, rMRGlu in the bilateral dorsolateral and ventrolateral PFC (BA 8, 9, 10), the right premotor cortex (BA 6), the right inferior parietal cortex (BA 40, cluster ranges into the occipital parts of BA 39 and BA 19), and the right superior temporal cortex (BA 13), were significantly positively correlated with the delayed recall performance in the RAVLT.

#### Recall consistency

Within the MDMA-group, rMRGlu only in the right DLPFC (BA 9) was correlated with recall consistency during the first five RAVLT trials.

#### Recognition

Within the MDMA-group, rMRGlu in the bilateral superior temporal cortices (BA 38), the right orbitofrontal cortex (gyrus rectus, BA 11), the left anterior hippocampus and parahippocampal gyrus (BA 28) and the left parieto-occipital part of the angular gyrus (BA 39) was associated with corrected recognition performance of list A (p(A)) of the RAVLT.

### Correlation of MDMA intake and resting rMRGlu

**Figure 3:**
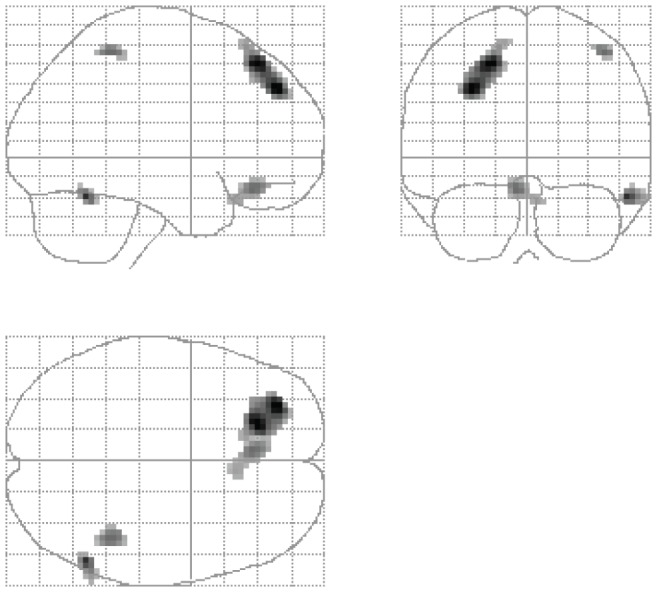
Brain regions in which decreased regional glucose metabolism was significantly correlated with high MDMA cumulative lifetime consumption in 19 recreational MDMA users (p< 0.005, uncorrected, cluster level >25 voxel).

To investigate the impact of cumulative MDMA intake across lifetime on structural alterations of the brain, we only investigated negative correlations between resting rMRGlu and drug dose (high consumption corresponding with decreased rMRGlu). Low rMRGlu was significantly correlated with high cumulative total dose of MDMA in the left dorsolateral (BA 8) and bilateral orbital and ventromedial PFC (BA 11, 25) as well as in the left inferior parietal cortex (BA 40) and the right lateral temporal cortex (BA 20, 37) (**Figures 3** **and** **4** **,** **Table 6**).

**Figure 4:**
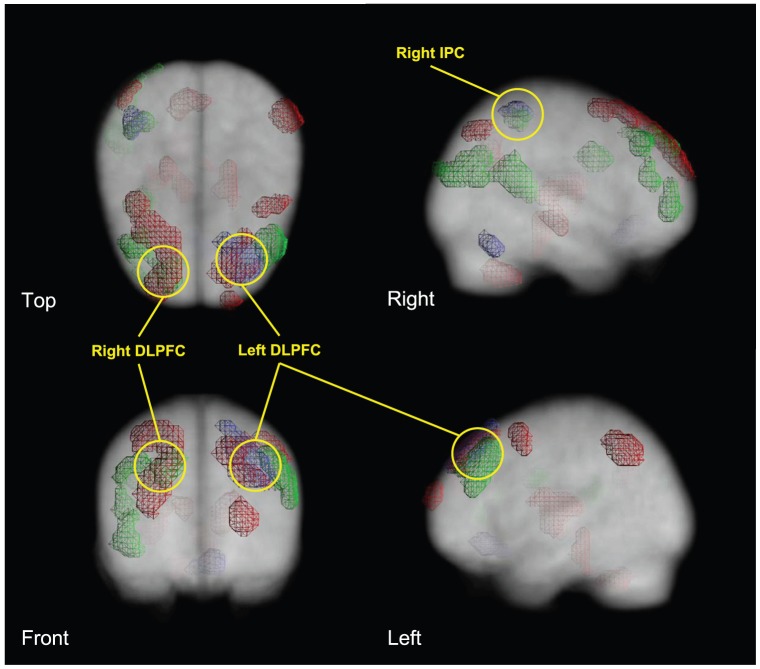
Brain regions with decreased regional glucose metabolism (red), and correlations of low memory performance (green) and high cumulative lifetime MDMA use with glucose metabolism (purple) in 19 recreational MDMA users (p<.005, uncorrected, cluster level >25 voxel, clusters projected on an ^18^FDG PET template drawn from the controls).

**Table 6:** Brain regions in which regional glucose metabolism was negatively correlated with cumulative lifetime dose of MDMA in 19 recreational MDMA users (MNI coordinates of maximum significant voxels, p<.005, uncorrected).

*z (df 17)*	Cluster size	MNI coordinates			P_uncorrected_		Hemisphere		Anatomical region	Brodmann area
	*Voxel*	*x,y,z (mm)*			*voxel-level*					
3.63	164^a^	−21	36	51		0.000		left	superior frontal gyrus	8
3.37	29	54	−57	−21		0.000		right	fusiform/inferior temporal gyrus	37/20
3.05	29	42	−42	57		0.001		right	inferior parietal lobule	40
3.04	66	−6	33	−18		0.001		left	medial frontal gyrus	11/25

a *Cluster significant at p<.05 (uncorrected).*

### Overlap of glucose hypometabolism, memory deficits, and drug intake

To investigate if there is an overlap between frontal hypometabolism, memory impairment and MDMA intake in MDMA users, we overlaid the significant clusters of the MDMA-control group contrast (rMRGlu hypometabolism in rest) with the correlation clusters between rMRGlu and delayed recall and between rMRGlu and MDMA intake in the PMOD 3.0 (PMOD Technologies, Zürich, Switzerland). All three significant cluster sets were projected on an FDG PET template drawn from the control group (**Figure 4**). Delayed recall was chosen because MDMA users showed the strongest impairment here.

Glucose hypometabolism of MDMA users strongly overlapped with positive correlations of rMRGlu and delayed recall in the bilateral DLPFC (superior/middle frontal gyrus; BA 8/9). Moreover, there was a triple overlap of MDMA intake, delayed recall, and hypometabolism in the left DLPFC. Finally, drug intake and delayed recall correlations with rMRGlu overlapped in right inferior parietal cortex (BA 40).

## Discussion

The aim of this study was to investigate the neurobiological correlates of memory deficits in currently abstinent recreational MDMA users. Using ^18^FDG-PET in rest, we demonstrated that MDMA users showed a significant reduction of cerebral glucose metabolism predominantly in the bilateral DLPFC and inferior parietal cortex, as well as the bilateral thalamus, right hippocampus, right precuneus, right cerebellum, and pons (at the level of raphe nuclei). In the group of MDMA users, learning and recall parameters were positively correlated with rMRGlu primarily in bilateral frontal and parietal brain regions, while word recognition was additionally related to rMRGlu in the right mediotemporal and bihemispheric lateral temporal cortex. In addition, cumulative lifetime dose of MDMA was negatively correlated with rMRGlu in the left DLPFC, bilateral ventromedial prefrontal and orbitofrontal cortex as well as left inferior parietal and right lateral temporal cortex. Importantly, glucose hypometabolism and rMRGlu correlations with recall deficits and cumulative MDMA intake altogether strongly overlapped within the left DLPFC of MDMA users, indicating that foremost drug-induced changes of the DLPFC might contribute to memory impairment in MDMA users.

Regarding affected brain structures in MDMA users, we replicated several previous findings reporting decreased glucose metabolism in the DLPFC [35] and hippocampus [32], [33] in MDMA users. Our results are also in line with previous studies showing changes of serotonergic radioligand binding in prefrontal and parietal regions [39]–[41], [44], hippocampus [39], and in the thalamus [37], [59] of MDMA users. In contrast, we did not find decreased rMRGlu in the amygdala and cingulate cortex or increased rMRGlu in the ventrolateral PFC as reported from the so far largest sample of MDMA users (n = 93 compared to n = 27 control subjects) [33]. However, our sample might have been underpowered to detect these additional changes.

Previous studies demonstrated that regional reduction of cerebral glucose metabolism might result from loss of neuronal connections and projections [60]–[62]. As such, the reductions of cortical rMRGlu in MDMA users might as well be explained by loss of neuronal connections and projections and thus be interpreted as a direct neurotoxic effect of the drug, as they were shown in many pervious animal studies [13], [63]. Interestingly, the thalamic hypometabolism of MDMA users shown here occurred in anatomical areas that are well connected to frontocortical regions presenting also hypometabolism [58]. Also the reduction of glucose metabolism at the brain stem level might be explained by lesions of the raphe nuclei as a previous study with non-human primates demonstrated pathological changes specifically in the dorsal raphe nucleus induced by MDMA [64]. Crucially, the symmetry of glucose hypometabolism in MDMA users, which occurred bilaterally in several regions such as the thalamus, frontal and prefrontal areas as well as the parietal cortex, confirms the validity and stability of the present results. Furthermore, it supports the argument of neurotoxicity, as neuronal damages would be expected to develop spatially nonspecific, thus equally distributed over both hemispheres [65].

Given that we have only investigated current users with a mean duration of abstinence of two to three weeks in a cross-sectional design, it can not be concluded if the reported alterations are lasting or transitory.

The results from this study confirm our hypothesis that hypometabolism in the frontal cortex and in the hippocampus is correlated with verbal memory deficits in recreational MDMA users. Within the MDMA group, worse working memory (supraspan) performance was correlated with low rMRGlu inferior parietal, premotor, dorsolateral, and left ventrolateral PFC. Given that the supraspan has a strong working memory component [66] this correlation pattern might indicate a specific or combined impairment of articulatory rehearsal processes (premotor cortex, ventrolateral PFC), of the phonological loop (inferior parietal lobule), and/or the central executive (DLPFC) in MDMA users [67]. Furthermore, MDMA users showed a correlation of recall consistency with rMRGlu in the right DLPFC, which is in line with the view the recall consistency represents an aspect of executive functions related to the DLPFC [68],

Within the MDMA group, total learning performance was associated with the right premotor cortex, bilateral dorsolateral prefrontal, and the left inferior frontal cortex. Therefore, learning deficits in MDMA users seem to be caused by dysfunction of frontal encoding or recall processes [69]. Interestingly, acute MDMA effects also seem to influence middle frontal gyrus (BA 10) activity leading to verbal memory encoding impairments [70].

The association of delayed recall and rMRGlu showed the broadest anatomical extension within the MDMA group. Delayed recall performance was positively correlated with the rMRGlu in a prefrontal cluster including the bilateral dorsolateral and ventrolateral PFC and right premotor cortex. Moreover, a parieto-temporal cluster including the right inferior parietal cortex and right superior temporal cortex appeared. Thus, prefrontal and parieto-temporal areas seem to be involved in the dysfunction of delayed recall in MDMA users, indicating disturbed memory formation into–or diminished recall from association cortices, beyond the shown working memory and frontal encoding and recall deficits [66].

Corrected recognition performance was associated with rMRGlu in bilateral superior temporal cortices, right orbitofrontal cortex (gyrus rectus), left anterior hippocampus and parahippocampal gyrus, and left parieto-occipital part of the angular gyrus. Therefore, particularly reduced recognition performance in MDMA users might be explained by hippocampal dysfunction [71]. However, also prefrontal and anterior temporal areas seem to be involved–areas that were previously linked with recognition in several PET studies [72].

Notably, most of the areas showing glucose hypometabolism in MDMA users are part of the two core brain networks: the resting-state default mode network (DMN; precuneus, lateral temporal cortex and hippocampus)[73] and its anti-correlated cognitive control network (CCN; DLPFC, parietal cortex)[74]. As cognitive impairment is assumed to reflect altered network connectivity between the DMN and the CCN, our findings might point to disrupted DMN-CCN connectivity, which should be further investigated using resting-state fMRI in MDMA users.

Taken together, the previously postulated dissociation of prefrontal and hippocampal functions as correlates of memory deficits in MDMA users, resulting from temporal or hippocampal dysfunction alone [21], [47], [49], [51], could not be confirmed. In our sample, verbal learning and recall deficits of MDMA users were correlated with glucose hypometabolism in the prefrontal, frontal and parietal cortex, while word recognition was additionally correlated with mediotemporal hypometabolism. Thus, our imaging data support the neuropsychology-based hypothesis of a combined fronto-parieto-mediotemporal dysfunction in abstinent MDMA users [27], [55]. This assumption is in accordance to the results of early animal studies showing that MDMA-induced denervation of 5-HT axons in the frontal cortex and hippocampus display a relatively low regeneration, thus indicating a high susceptibility of these structures for MDMA neurotoxicity [13], [75], [76].

The cumulative total dose of MDMA was negatively correlated with rMRGlu in the left dorsolateral and bilateral orbital and ventromedial PFC, left inferior parietal, and right lateral temporal cortex. This finding is largely in line with a recent study showing significant correlation of the severity of MDMA, heroin, alcohol, and cannabis use with rMRGlu in the DLPFC and lateral temporal cortex of polydrug users [35].

A limitation of this study is the use of subjective reports for assessing the extent of drug consumption. The reliability of these reports may be questionable, as they depend on memory function and are probably influenced by anticipated social expectancies. A solution of this problem would be a toxicological hair analysis, which was not available for us at that time. In this regard, our urine drug screenings are only of restricted advantage, as water soluble substances such as MDMA can only be analyzed until few days post exposure [77]. However, in a study that addressed this problem, a concordance of 91.3 % was found between subjective reports of drug consumption and toxicological hair analyzes of MDMA users, indicating an acceptable reliability of such reports [78]. Collectively, it is a pertinent unsolved problems of neurotoxicological research, that an objective method to determine the cumulative lifetime drug consumption is not available to date [79]. Another key issue of human neurotoxicity studies in illicit drug users is the high prevalence of polytoxic drug use in this population. Although, this study included only subjects with a predominant use of MDMA, these participants also used other drugs. A retrospective differentiation of single substance effects is difficult, as the use of the different substances is highly intercorrelated. In addition, potential protective or potentiating effects of substance combinations on neurotoxicity limit the interpretation of such results. Moreover, tobacco use was significantly more pronounced in MDMA users compared to controls. Given, that nicotine has considerable effects on brain networks and cognitive functions [80], [81], the modulating effect of nicotine on our results is not entirely clear although introduction of smoking as a covariate into the group comparisons did not change the main results. Finally, the abstinence duration from the different drugs was highly variable between subjects; however, the abstinence duration of MDMA, cannabis, cocaine, amphetamine and hallucinogens was not systematically correlated with rMRGlu in the user group.

The availability of biological markers for the central serotonergic system such as 5-HT or 5-HIAA CSF or plasma concentrations would have been of advantage for the interpretation of our study results. However, to date such markers have only low validity and reliability [82]. Using a specific serotonergic radioligand might have been of additional interest, as alterations of the cerebral glucose metabolism only reflect global functioning of neuronal tissue. Also in this case, the reliability and specificity of in particular 5-HTT PET ligands seem questionable, as they are not able to reflect global serotonergic functioning [83], [84]. Recently, we developed a novel method employing ^18^F-altanserin PET in combination with a dexfenfluramine challenge in order to measure 5-HT release capacity [85], which now provides the first functional measure for the 5-HT system in humans. Application of this method to MDMA users will allow the first functional investigation of the impact of chronic MDMA intake on 5-HT release capacity.

### Conclusion

Our ^18^FDG PET and neuropsychological data show that verbal memory deficits are correlated with glucose hypometabolism in dorsolateral prefrontal and parietal areas of the brains of abstinent MDMA users. This is especially true for global learning performance and delayed recall, while recognition is additionally associated with mediotemporal hypometabolism. Frontal and parietal areas show the highest overlap of glucose hypometabolism with impaired memory function and cumulative drug intake. In conclusion, memory deficits of MDMA users seem to arise from combined fronto-parieto-mediotemporal dysfunction.

## Methods

### Ethic Statement

The study was approved by the Ethics Committee of the Medical Faculty of the University of Bonn. After being informed of the aim of the study by written and oral description, all participants gave written informed-consent statements. The study has been conducted according to the principles expressed in the Declaration of Helsinki.

### Participants

Nineteen male, chronic but currently abstinent users of MDMA and 19 subjects with no history of illicit drug use were studied (all of Caucasian ethnicity). All participants were recruited by advertisements in a techno music magazine. Subjects of the MDMA group were required to have used MDMA at least 50 times in lifetime and over a period of at least one year. In addition, the use of MDMA clearly had to dominate the consumption of any other psychotropic drug and participants should especially have no substantial previous use of other amphetamine derivatives (e.g., methamphetamine) or cocaine. PET scans and neuropsychological assessments were carried out when participants were abstinent for at least three days. Inclusion criteria for both groups included negative drug urine toxicology. Exclusion criteria for both groups included use of any psychotropic medication, a present psychiatric disorder, a family history of psychiatric disorders, as well as a severe somatic disease. None of the participants reported a history of migraine, epilepsy, or craniocerebral trauma.

### Procedure

On the first day the neuropsychological test battery was assessed in the afternoon (2 p.m.), while on the subsequent day the PET scan was carried out in the morning (8 a.m.). For the estimation of verbal intellectual performance, the *Mehrfachwahl-Wortschatz-Intelligenztest* was used [86]. To control for psychiatric disorders, we conducted a *Structured Clinical Interview* (SCID-I) interview according to *Diagnostic and Statistical Manual* IV (DSM-IV) procedures. Drug history and present pattern of psychotropic drug consumption was assessed by the *Interview for Psychotropic Drug Consumption* [30]. Afterwards a neuropsychological test battery was applied, including the RAVLT [27], [55]. The whole battery took about 120 minutes; including breaks as needed, and was generally well tolerated by the participants. During the psychiatric and neuropsychological assessment, the subjects could ask for a break at any time.

### Verbal memory assessment

Employment of the *Verbaler Lern- und Merkfähigkeitstest* [66]–a German version of the RAVLT [87]–has been described in detail before [27]. In brief, the instrument consists of a word list A of 15 nouns (learning list), a second word list B of 15 different nouns (interference list) and a third list C of 50 nouns which includes all words of lists A and B as well as 20 new, but semantically- and phonetically-related words (recognition list). The dependent variables used here are: supraspan (trial 1), learning performance (Σ trials 1–5), recall consistency in percent (according to [88]), delayed recall (trial 7 after 30 min), and adjusted recognition performance (*p*(*A*)_list A_ according to [89]). *p*(*A*) is comparable to the discrimination performance (d-prime) of the signal detection theory [90].

### Positron Emission Tomography

On the second day, ^18^FDG PET scans in rest were conducted according to the *European Association of Nuclear Medicine Procedures Guidelines for Brain Imaging using ^18^FDG-*PET (*Version I*)[91], using a whole-body, high-resolution positron emission tomograph (Siemens/ ECAT EXACT 47, with 5.8×5.8×5 mm) with ^18^FDG as radiotracer. A ten-minute emission scan was started 30 minutes after injection of 296–370 MBq ^18^FDG. This was followed by a five-minute transmission scan using ^68^germanium. Image reconstruction was performed due to filtered back projection using a Hannig-Filter (cut-off frequency of 0.5 cycles), thus leading to 47 transversal layers of 128×128 pixels each and voxel sizes of 2.059×2.059×3.375 mm. Blood glucose levels were assessed anterior to the injection of the tracer. Arterialized blood sampling was used to measure ^18^FDG in plasma. Subjects were scanned once, under resting conditions (light dimmed, subjects lying, and eyes closed) during 8:00 and 9:30 a.m., each.

### Data Analysis

Age, verbal IQ, years of education, and memory parameters were analyzed using t-tests for independent samples (SPSS 12, Chicago, IL). Handedness and the ratio of smokers and non-smokers were calculated using χ^2^-tests with Yates correction.

PET data were analyzed using Statistical Parametric Mapping 5 (SPM5; Wellcome Functional Imaging Laboratory, London, UK). According to the convention of the *Montreal Neurological Institution* (MNI), the images were spatially normalized using the PET template provided in SPM5 and subsequently smoothed with an isotropic Gaussian kernel to 12×12×12 mm full width at half maximum (FWHM). The normalized and smoothed images were adjusted by a proportional scaling (mean to 50, threshold masking 0.8) for the individual global differences in cerebral glucose metabolism. For voxel-wise comparisons, t-test for independent samples was used. Voxel-wise correlations were performed to assess the relationship between changes in rMRGlu and changes in individual cognitive performance within each group. Because we would have liked to avoid “voodoo correlations” we did not correlate memory performance with metabolic rates within clusters showing significant rMRGlu differences between both groups [92]. Significance threshold was set at p<0.005 (uncorrected, cluster level >25 voxels). Statistical maps were projected on an MRI T1 structural image template provided by SPM5.

## References

[1] Christophersen AS (2000) Amphetamine designer drugs-an overview and epidemiology. Toxicol Lett 112–113: 127–131. 10.1016/s0378-4274(99)00205-2 10720721

[2] Sumnall HR, Cole JC, Jerome L (2006) The varieties of ecstatic experience: an exploration of the subjective experiences of ecstasy. J Psychopharmacol 20: 670–682. 16401654 10.1177/0269881106060764

[3] European Monitoring Centre for Drugs and Drug Addiction (EMCDDA) (2012) Annual report on the state of the drugs problem in Europe. Lisbon: European Monitoring Centre for Drugs and Drug Addiction

[4] United Nations Office on Drugs and Crime (UNODC) (2012) World Drug Report 2012. Vienna: United Nations.

[5] Rudnick G, Wall SC (1992) The molecular mechanism of "ecstasy" [3,4-methylenedioxy-methamphetamine (MDMA)]: serotonin transporters are targets for MDMA-induced serotonin release. Proc Natl Acad Sci U S A 89: 1817–1821. 1347426 10.1073/pnas.89.5.1817 PMC48544

[6] Green AR, Mechan AO, Elliott JM, O'Shea E, Colado MI (2003) The pharmacology and clinical pharmacology of 3,4-methylenedioxymethamphetamine (MDMA, "ecstasy"). Pharmacol Rev 55: 463–508. 12869661 10.1124/pr.55.3.3

[7] Hagino Y, Takamatsu Y, Yamamoto H, Iwamura T, Murphy DL, et al (2011) Effects of MDMA on Extracellular Dopamine and Serotonin Levels in Mice Lacking Dopamine and/or Serotonin Transporters. Curr Neuropharmacol 9: 91–95. 21886569 10.2174/157015911795017254 PMC3137209

[8] Anneken JH, Gudelsky GA (2012) MDMA produces a delayed and sustained increase in the extracellular concentration of glutamate in the rat hippocampus. Neuropharmacology 63: 1022–1027. 22842073 10.1016/j.neuropharm.2012.07.026 PMC3437747

[9] Parrott AC (2009) Cortisol and 3,4-methylenedioxymethamphetamine: neurohormonal aspects of bioenergetic stress in ecstasy users. Neuropsychobiology 60: 148–158. 19893332 10.1159/000253551 PMC2826870

[10] Lyles J, Cadet JL (2003) Methylenedioxymethamphetamine (MDMA, Ecstasy) neurotoxicity: cellular and molecular mechanisms. Brain Res Brain Res Rev 42: 155–168. 12738056 10.1016/s0165-0173(03)00173-5

[11] Steinkellner T, Freissmuth M, Sitte HH, Montgomery T (2011) The ugly side of amphetamines: short- and long-term toxicity of 3,4-methylenedioxymethamphetamine (MDMA, 'Ecstasy'), methamphetamine and D-amphetamine. Biol Chem 392: 103–115. 21194370 10.1515/BC.2011.016 PMC4497800

[12] Ali SF, Newport GD, Scallet AC, Binienda Z, Ferguson SA, et al (1993) Oral administration of 3,4-methylenedioxymethamphetamine (MDMA) produces selective serotonergic depletion in the nonhuman primate. Neurotoxicol Teratol 15: 91–96. 7685472 10.1016/0892-0362(93)90067-x

[13] Hatzidimitriou G, McCann UD, Ricaurte GA (1999) Altered serotonin innervation patterns in the forebrain of monkeys treated with (+/−)3,4-methylenedioxymethamphetamine seven years previously: factors influencing abnormal recovery. J Neurosci 19: 5096–5107. 10366642 10.1523/JNEUROSCI.19-12-05096.1999 PMC6782677

[14] Insel TR, Battaglia G, Johannessen JN, Marra S, De Souza EB (1989) 3,4-Methylenedioxymethamphetamine ("ecstasy") selectively destroys brain serotonin terminals in rhesus monkeys. J Pharmacol Exp Ther 249: 713–720. 2471824

[15] Scheffel U, Szabo Z, Mathews WB, Finley PA, Dannals RF, et al (1998) In vivo detection of short- and long-term MDMA neurotoxicity–a positron emission tomography study in the living baboon brain. Synapse 29: 183–192. 9593108 10.1002/(SICI)1098-2396(199806)29:2<183::AID-SYN9>3.0.CO;2-3

[16] Wilson MA, Ricaurte GA, Molliver ME (1989) Distinct morphologic classes of serotonergic axons in primates exhibit differential vulnerability to the psychotropic drug 3,4-methylenedioxymethamphetamine. Neuroscience 28: 121–137. 2761687 10.1016/0306-4522(89)90237-6

[17] McCann UD, Ridenour A, Shaham Y, Ricaurte GA (1994) Serotonin neurotoxicity after (+/−)3,4-methylenedioxymethamphetamine (MDMA; "Ecstasy"): a controlled study in humans. Neuropsychopharmacology 10: 129–138. 7517677 10.1038/npp.1994.15

[18] Ricaurte GA, Finnegan KT, Irwin I, Langston JW (1990) Aminergic metabolites in cerebrospinal fluid of humans previously exposed to MDMA: preliminary observations. Ann N Y Acad Sci 600: 699–708; discussion 708–610. 1701292 10.1111/j.1749-6632.1990.tb16919.x

[19] Bhattachary S, Powell JH (2001) Recreational use of 3,4-methylenedioxymethamphetamine (MDMA) or 'ecstasy': evidence for cognitive impairment. Psychol Med 31: 647–658. 11352367 10.1017/s0033291701003828

[20] Fox HC, Toplis AS, Turner JJ, Parrott AC (2001) Auditory verbal learning in drug-free Ecstasy polydrug users. Hum Psychopharmacol 16: 613–618. 12404541 10.1002/hup.344

[21] Gouzoulis-Mayfrank E, Thimm B, Rezk M, Hensen G, Daumann J (2003) Memory impairment suggests hippocampal dysfunction in abstinent ecstasy users. Prog Neuropsychopharmacol Biol Psychiatry 27: 819–827. 12921915 10.1016/S0278-5846(03)00114-3

[22] Rodgers J (2000) Cognitive performance amongst recreational users of "ecstasy". Psychopharmacology (Berl) 151: 19–24. 10958112 10.1007/s002130000467

[23] Wareing M, Fisk JE, Murphy P, Montgomery C (2004) Verbal working memory deficits in current and previous users of MDMA. Hum Psychopharmacol 19: 225–234. 15181650 10.1002/hup.586

[24] Wareing M, Fisk JE, Murphy P, Montgomery C (2005) Visuo-spatial working memory deficits in current and former users of MDMA ('ecstasy'). Hum Psychopharmacol 20: 115–123. 15641126 10.1002/hup.670

[25] McCann UD, Mertl M, Eligulashvili V, Ricaurte GA (1999) Cognitive performance in (+/−) 3,4-methylenedioxymethamphetamine (MDMA, "ecstasy") users: a controlled study. Psychopharmacology (Berl) 143: 417–425. 10367560 10.1007/s002130050967

[26] Morgan MJ (1999) Memory deficits associated with recreational use of "ecstasy" (MDMA). Psychopharmacology (Berl) 141: 30–36. 9952062 10.1007/s002130050803

[27] Quednow BB, Jessen F, Kuhn KU, Maier W, Daum I, et al (2006) Memory deficits in abstinent MDMA (ecstasy) users: neuropsychological evidence of frontal dysfunction. J Psychopharmacol 20: 373–384. 16574711 10.1177/0269881106061200

[28] Bolla KI, McCann UD, Ricaurte GA (1998) Memory impairment in abstinent MDMA ("Ecstasy") users. Neurology 51: 1532–1537. 9855498 10.1212/wnl.51.6.1532

[29] Croft RJ, Klugman A, Baldeweg T, Gruzelier JH (2001) Electrophysiological evidence of serotonergic impairment in long-term MDMA ("ecstasy") users. Am J Psychiatry 158: 1687–1692. 11579003 10.1176/appi.ajp.158.10.1687

[30] Quednow BB, Kuhn KU, Hoenig K, Maier W, Wagner M (2004) Prepulse inhibition and habituation of acoustic startle response in male MDMA ('ecstasy') users, cannabis users, and healthy controls. Neuropsychopharmacology 29: 982–990. 14970829 10.1038/sj.npp.1300396

[31] Tuchtenhagen F, Daumann J, Norra C, Gobbele R, Becker S, et al (2000) High intensity dependence of auditory evoked dipole source activity indicates decreased serotonergic activity in abstinent ecstasy (MDMA) users. Neuropsychopharmacology 22: 608–617. 10788760 10.1016/S0893-133X(99)00140-2

[32] Obrocki J, Buchert R, Vaterlein O, Thomasius R, Beyer W, et al (1999) Ecstasy–long-term effects on the human central nervous system revealed by positron emission tomography. Br J Psychiatry 175: 186–188. 10627804 10.1192/bjp.175.2.186

[33] Buchert R, Obrocki J, Thomasius R, Vaterlein O, Petersen K, et al (2001) Long-term effects of 'ecstasy' abuse on the human brain studied by FDG PET. Nucl Med Commun 22: 889–897. 11473208 10.1097/00006231-200108000-00007

[34] Obrocki J, Schmoldt A, Buchert R, Andresen B, Petersen K, et al (2002) Specific neurotoxicity of chronic use of ecstasy. Toxicol Lett 127: 285–297. 12052669 10.1016/s0378-4274(01)00511-2

[35] Moreno-Lopez L, Stamatakis EA, Fernandez-Serrano MJ, Gomez-Rio M, Rodriguez-Fernandez A, et al (2012) Neural correlates of the severity of cocaine, heroin, alcohol, MDMA and cannabis use in polysubstance abusers: a resting-PET brain metabolism study. PLoS One 7: e39830. 22768136 10.1371/journal.pone.0039830 PMC3387209

[36] Buchert R, Thiele F, Thomasius R, Wilke F, Petersen K, et al (2007) Ecstasy-induced reduction of the availability of the brain serotonin transporter as revealed by [11C](+)McN5652-PET and the multi-linear reference tissue model: loss of transporters or artifact of tracer kinetic modelling? J Psychopharmacol 21: 628–634. 17092972 10.1177/0269881106071975

[37] Buchert R, Thomasius R, Nebeling B, Petersen K, Obrocki J, et al (2003) Long-term effects of "ecstasy" use on serotonin transporters of the brain investigated by PET. J Nucl Med 44: 375–384. 12621003

[38] Buchert R, Thomasius R, Wilke F, Petersen K, Nebeling B, et al (2004) A voxel-based PET investigation of the long-term effects of "Ecstasy" consumption on brain serotonin transporters. Am J Psychiatry 161: 1181–1189. 15229049 10.1176/appi.ajp.161.7.1181

[39] Kish SJ, Lerch J, Furukawa Y, Tong J, McCluskey T, et al (2010) Decreased cerebral cortical serotonin transporter binding in ecstasy users: a positron emission tomography/[(11)C]DASB and structural brain imaging study. Brain 133: 1779–1797. 20483717 10.1093/brain/awq103 PMC2912692

[40] McCann UD, Szabo Z, Scheffel U, Dannals RF, Ricaurte GA (1998) Positron emission tomographic evidence of toxic effect of MDMA ("Ecstasy") on brain serotonin neurons in human beings. Lancet 352: 1433–1437. 9807990 10.1016/s0140-6736(98)04329-3

[41] McCann UD, Szabo Z, Seckin E, Rosenblatt P, Mathews WB, et al (2005) Quantitative PET studies of the serotonin transporter in MDMA users and controls using [11C]McN5652 and [11C]DASB. Neuropsychopharmacology 30: 1741–1750. 15841106 10.1038/sj.npp.1300736 PMC2034411

[42] Erritzoe D, Frokjaer VG, Holst KK, Christoffersen M, Johansen SS, et al (2011) In vivo imaging of cerebral serotonin transporter and serotonin(2A) receptor binding in 3,4-methylenedioxymethamphetamine (MDMA or "ecstasy") and hallucinogen users. Arch Gen Psychiatry 68: 562–576. 21646575 10.1001/archgenpsychiatry.2011.56

[43] Selvaraj S, Hoshi R, Bhagwagar Z, Murthy NV, Hinz R, et al (2009) Brain serotonin transporter binding in former users of MDMA ('ecstasy'). Br J Psychiatry 194: 355–359. 19336788 10.1192/bjp.bp.108.050344

[44] Reneman L, Endert E, de Bruin K, Lavalaye J, Feenstra MG, et al (2002) The acute and chronic effects of MDMA ("ecstasy") on cortical 5-HT2A receptors in rat and human brain. Neuropsychopharmacology 26: 387–396. 11850153 10.1016/S0893-133X(01)00366-9

[45] Di Iorio CR, Watkins TJ, Dietrich MS, Cao A, Blackford JU, et al (2011) Evidence for Chronically Altered Serotonin Function in the Cerebral Cortex of Female 3,4-Methylenedioxymethamphetamine Polydrug Users. Arch Gen Psychiatry 10.1001/archgenpsychiatry.2011.156 PMC3538835 22147810

[46] Urban NB, Girgis RR, Talbot PS, Kegeles LS, Xu X, et al (2012) Sustained recreational use of ecstasy is associated with altered pre and postsynaptic markers of serotonin transmission in neocortical areas: a PET study with [(1)(1)C]DASB and [(1)(1)C]MDL 100907. Neuropsychopharmacology 37: 1465–1473. 22353758 10.1038/npp.2011.332 PMC3327851

[47] Fox HC, McLean A, Turner JJ, Parrott AC, Rogers R, et al (2002) Neuropsychological evidence of a relatively selective profile of temporal dysfunction in drug-free MDMA ("ecstasy") polydrug users. Psychopharmacology (Berl) 162: 203–214. 12110998 10.1007/s00213-002-1071-9

[48] Becker B, Wagner D, Koester P, Bender K, Kabbasch C, et al (2012) Memory-related hippocampal functioning in ecstasy and amphetamine users: A prospective fMRI study. Psychopharmacology (Berl) 10.1007/s00213-012-2873-z 23001254

[49] Daumann J, Fischermann T, Heekeren K, Henke K, Thron A, et al (2005) Memory-related hippocampal dysfunction in poly-drug ecstasy (3,4-methylenedioxymethamphetamine) users. Psychopharmacology (Berl) 180: 607–611. 15372137 10.1007/s00213-004-2002-8

[50] Daumann J Jr, Fischermann T, Heekeren K, Thron A, Gouzoulis-Mayfrank E (2004) Neural mechanisms of working memory in ecstasy (MDMA) users who continue or discontinue ecstasy and amphetamine use: evidence from an 18-month longitudinal functional magnetic resonance imaging study. Biol Psychiatry 56: 349–355. 15336517 10.1016/j.biopsych.2004.06.011

[51] Jacobsen LK, Mencl WE, Pugh KR, Skudlarski P, Krystal JH (2004) Preliminary evidence of hippocampal dysfunction in adolescent MDMA ("ecstasy") users: possible relationship to neurotoxic effects. Psychopharmacology (Berl) 173: 383–390. 14647960 10.1007/s00213-003-1679-4

[52] Moeller FG, Steinberg JL, Dougherty DM, Narayana PA, Kramer LA, et al (2004) Functional MRI study of working memory in MDMA users. Psychopharmacology (Berl) 177: 185–194. 15221201 10.1007/s00213-004-1908-5

[53] Daumann J, Fimm B, Willmes K, Thron A, Gouzoulis-Mayfrank E (2003) Cerebral activation in abstinent ecstasy (MDMA) users during a working memory task: a functional magnetic resonance imaging (fMRI) study. Brain Res Cogn Brain Res 16: 479–487. 12706227 10.1016/s0926-6410(03)00075-2

[54] Roberts GM, Nestor L, Garavan H (2009) Learning and memory deficits in ecstasy users and their neural correlates during a face-learning task. Brain Res 1292: 71–81. 19631624 10.1016/j.brainres.2009.07.040

[55] Quednow BB, Kuhn KU, Hoppe C, Westheide J, Maier W, et al (2007) Elevated impulsivity and impaired decision-making cognition in heavy users of MDMA ("Ecstasy"). Psychopharmacology (Berl) 189: 517–530. 16425060 10.1007/s00213-005-0256-4

[56] Valdes IH, Steinberg JL, Narayana PA, Kramer LA, Dougherty DM, et al (2006) Impulsivity and BOLD fMRI activation in MDMA users and healthy control subjects. Psychiatry Res 147: 239–242. 16890410 10.1016/j.pscychresns.2006.01.014

[57] Kalechstein AD, De La Garza R, 2nd, Mahoney JJ, 3rd, Fantegrossi WE, Newton TF (2007) MDMA use and neurocognition: a meta-analytic review. Psychopharmacology (Berl) 189: 531–537. 17082969 10.1007/s00213-006-0601-2

[58] Behrens TE, Johansen-Berg H, Woolrich MW, Smith SM, Wheeler-Kingshott CA, et al (2003) Non-invasive mapping of connections between human thalamus and cortex using diffusion imaging. Nat Neurosci 6: 750–757. 12808459 10.1038/nn1075

[59] de Win MM, Jager G, Booij J, Reneman L, Schilt T, et al (2008) Neurotoxic effects of ecstasy on the thalamus. Br J Psychiatry 193: 289–296. 18827290 10.1192/bjp.bp.106.035089

[60] Hampel H, Teipel SJ, Alexander GE, Pogarell O, Rapoport SI, et al (2002) In vivo imaging of region and cell type specific neocortical neurodegeneration in Alzheimer's disease. Perspectives of MRI derived corpus callosum measurement for mapping disease progression and effects of therapy. Evidence from studies with MRI, EEG and PET. J Neural Transm 109: 837–855. 12111472 10.1007/s007020200069

[61] Kew JJ, Brooks DJ, Passingham RE, Rothwell JC, Frackowiak RS, et al (1994) Cortical function in progressive lower motor neuron disorders and amyotrophic lateral sclerosis: a comparative PET study. Neurology 44: 1101–1110. 8208409 10.1212/wnl.44.6.1101

[62] Millien I, Blaizot X, Giffard C, Mezenge F, Insausti R, et al (2002) Brain glucose hypometabolism after perirhinal lesions in baboons: implications for Alzheimer disease and aging. J Cereb Blood Flow Metab 22: 1248–1261. 12368664 10.1097/01.WCB.0000037997.34930.67

[63] Dzietko M, Sifringer M, Klaus J, Endesfelder S, Brait D, et al (2010) Neurotoxic effects of MDMA (ecstasy) on the developing rodent brain. Dev Neurosci 32: 197–207. 20616555 10.1159/000313473

[64] Ricaurte GA, Forno LS, Wilson MA, DeLanney LE, Irwin I, et al (1988) (+/−)3,4-Methylenedioxymethamphetamine selectively damages central serotonergic neurons in nonhuman primates. Jama 260: 51–55. 2454332

[65] Rosenberg NL (1995) Basic principles of clinical neurotoxicology. In: Chang LW, Slikker W, Jr., editors. Neurotoxicology-Approaches and methods: Academic press.

[66] Helmstaedter C, Lendt M, Lux S (2001) Verbaler Lern- und Merkfähigkeitstest, Manual. Göttingen: Beltz

[67] Baddeley A (2003) Working memory: looking back and looking forward. Nat Rev Neurosci 4: 829–839. 14523382 10.1038/nrn1201

[68] Alexander MP, Stuss DT, Fansabedian N (2003) California Verbal Learning Test: performance by patients with focal frontal and non-frontal lesions. Brain 126: 1493–1503. 12764068 10.1093/brain/awg128

[69] Ranganath C, Johnson MK, D'Esposito M (2003) Prefrontal activity associated with working memory and episodic long-term memory. Neuropsychologia 41: 378–389. 12457762 10.1016/s0028-3932(02)00169-0

[70] Kuypers KP, Wingen M, Heinecke A, Formisano E, Ramaekers JG (2011) MDMA intoxication and verbal memory performance: a placebo-controlled pharmaco-MRI study. J Psychopharmacol 25: 1053–1061. 21616977 10.1177/0269881111405361

[71] Aggleton JP, Brown MW (1999) Episodic memory, amnesia, and the hippocampal-anterior thalamic axis. Behav Brain Sci 22: 425–444; discussion 444–489. 11301518

[72] Tulving E, Habib R, Nyberg L, Lepage M, McIntosh AR (1999) Positron emission tomography correlations in and beyond medial temporal lobes. Hippocampus 9: 71–82. 10088902 10.1002/(SICI)1098-1063(1999)9:1<71::AID-HIPO8>3.0.CO;2-F

[73] Buckner RL, Andrews-Hanna JR, Schacter DL (2008) The brain's default network: anatomy, function, and relevance to disease. Ann N Y Acad Sci 1124: 1–38. 18400922 10.1196/annals.1440.011

[74] Niendam TA, Laird AR, Ray KL, Dean YM, Glahn DC, et al (2012) Meta-analytic evidence for a superordinate cognitive control network subserving diverse executive functions. Cogn Affect Behav Neurosci 12: 241–268. 22282036 10.3758/s13415-011-0083-5 PMC3660731

[75] Fischer C, Hatzidimitriou G, Wlos J, Katz J, Ricaurte G (1995) Reorganization of ascending 5-HT axon projections in animals previously exposed to the recreational drug (+/−)3,4-methylenedioxymethamphetamine (MDMA, "ecstasy"). J Neurosci 15: 5476–5485. 7643196 10.1523/JNEUROSCI.15-08-05476.1995 PMC6577639

[76] Ricaurte GA, Martello AL, Katz JL, Martello MB (1992) Lasting effects of (+−)-3,4-methylenedioxymethamphetamine (MDMA) on central serotonergic neurons in nonhuman primates: neurochemical observations. J Pharmacol Exp Ther 261: 616–622. 1374470

[77] Ensslin HK, Kovar KA, Maurer HH (1996) Toxicological detection of the designer drug 3,4-methylenedioxyethylamphetamine (MDE, "Eve") and its metabolites in urine by gas chromatography-mass spectrometry and fluorescence polarization immunoassay. J Chromatogr B Biomed Appl 683: 189–197. 8891915 10.1016/0378-4347(96)00129-6

[78] Stuerenburg HJ, Petersen K, Baumer T, Rosenkranz M, Buhmann C, et al (2002) Plasma concentrations of 5-HT, 5-HIAA, norepinephrine, epinephrine and dopamine in ecstasy users. Neuro Endocrinol Lett 23: 259–261. 12080289

[79] Curran HV (2000) Is MDMA ('Ecstasy') neurotoxic in humans? An overview of evidence and of methodological problems in research. Neuropsychobiology 42: 34–41. 10867554 10.1159/000026668

[80] Newhouse PA, Potter AS, Dumas JA, Thiel CM (2011) Functional brain imaging of nicotinic effects on higher cognitive processes. Biochem Pharmacol 82: 943–951. 21684262 10.1016/j.bcp.2011.06.008 PMC3162085

[81] Wylie KP, Rojas DC, Tanabe J, Martin LF, Tregellas JR (2012) Nicotine increases brain functional network efficiency. Neuroimage 63: 73–80. 22796985 10.1016/j.neuroimage.2012.06.079 PMC3429645

[82] Taffe MA, Davis SA, Yuan J, Schroeder R, Hatzidimitriou G, et al (2002) Cognitive performance of MDMA-treated rhesus monkeys: sensitivity to serotonergic challenge. Neuropsychopharmacology 27: 993–1005. 12464456 10.1016/S0893-133X(02)00380-9

[83] Hoyer D, Hannon JP, Martin GR (2002) Molecular, pharmacological and functional diversity of 5-HT receptors. Pharmacol Biochem Behav 71: 533–554. 11888546 10.1016/s0091-3057(01)00746-8

[84] Kish SJ (2002) How strong is the evidence that brain serotonin neurons are damaged in human users of ecstasy? Pharmacol Biochem Behav 71: 845–855. 11888575 10.1016/s0091-3057(01)00708-0

[85] Quednow BB, Treyer V, Hasler F, Dorig N, Wyss MT, et al (2012) Assessment of serotonin release capacity in the human brain using dexfenfluramine challenge and [18F]altanserin positron emission tomography. Neuroimage 59: 3922–3932. 21996132 10.1016/j.neuroimage.2011.09.045

[86] Lehrl S (2005) Mehrfachwahl-Wortschatz-Intelligenztest: MWT-B. Balingen

[87] Rey A (1964) L'examen de Clinique en Psychologie. Paris: Presses Universitaire de France.

[88] Delis DC, Kramer J, Kaplan E, Ober BA (1987) California Verbal Learning Test, Adult Verision Manual. San Antonio, TX: Psychological Cooperation.

[89] Forrester G, Geffen G (1991) Performance measures of 7- to 15-year-old children on the Auditory Verbal Learning Test. The Clinical Neuropsychologist 5: 345–359. 10.1080/13854049008401496 29022439

[90] Green DM, Swets JA (1966) Signal Detection Theory and Psychophysics. London: J. Wiley.

[91] Bartenstein P, Asenbaum S, Catafau A, Halldin C, Pilowski L, et al (2002) European Association of Nuclear Medicine procedure guidelines for brain imaging using [(18)F]FDG. Eur J Nucl Med Mol Imaging 29: BP43–48. 12436498

[92] Vul E, Pashler H (2012) Voodoo and circularity errors. Neuroimage 62: 945–948. 22270348 10.1016/j.neuroimage.2012.01.027

